# Mitochondrial Ferritin Deletion Exacerbates *β*-Amyloid-Induced Neurotoxicity in Mice

**DOI:** 10.1155/2017/1020357

**Published:** 2017-01-16

**Authors:** Peina Wang, Qiong Wu, Wenyue Wu, Haiyan Li, Yuetong Guo, Peng Yu, Guofen Gao, Zhenhua Shi, Baolu Zhao, Yan-Zhong Chang

**Affiliations:** Laboratory of Molecular Iron Metabolism, The Key Laboratory of Animal Physiology, Biochemistry and Molecular Biology of Hebei Province, College of Life Science, Hebei Normal University, Shijiazhuang, Hebei 050024, China

## Abstract

Mitochondrial ferritin (FtMt) is a mitochondrial iron storage protein which protects mitochondria from iron-induced oxidative damage. Our previous studies indicate that FtMt attenuates *β*-amyloid- and 6-hydroxydopamine-induced neurotoxicity in SH-SY5Y cells. To explore the protective effects of FtMt on *β*-amyloid-induced memory impairment and neuronal apoptosis and the mechanisms involved, 10-month-old wild-type and* Ftmt* knockout mice were infused intracerebroventricularly (ICV) with A*β*_25–35_ to establish an Alzheimer's disease model. Knockout of* Ftmt* significantly exacerbated A*β*_25–35_-induced learning and memory impairment. The Bcl-2/Bax ratio in mouse hippocampi was decreased and the levels of cleaved caspase-3 and PARP were increased. The number of neuronal cells undergoing apoptosis in the hippocampus was also increased in* Ftmt* knockout mice. In addition, the levels of L-ferritin and FPN1 in the hippocampus were raised, and the expression of TfR1 was decreased. Increased MDA levels were also detected in* Ftmt* knockout mice treated with A*β*_25–35_. In conclusion, this study demonstrated that the neurological impairment induced by A*β*_25–35_ was exacerbated in* Ftmt* knockout mice and that this may relate to increased levels of oxidative stress.

## 1. Introduction

Alzheimer's disease (AD) is a multifaceted neurodegenerative disease of the elderly which is characterized by neuronal loss, neuroinflammation, and progressive memory and cognitive impairment [1]. Many pathogenic factors are involved in the neuropathology, including accumulation of *β*-amyloid (A*β*), oxidative stress, inflammation, and metal deposition [2]. A*β* is considered to be a major factor in the pathophysiological mechanisms underlying AD and has been shown to directly induce neuronal cell death [3]. Thus, A*β* is a useful tool for establishing AD models and investigating the mechanisms involved in AD pathogenesis [4]. A*β*_25–35_ is an 11-amino acid fragment located in the hydrophobic functional domain of the C-terminal region of A*β*_1–42_ [5]. Single administration of A*β*_25–35_ into the lateral ventricles of mice or rats impairs memory and induces neurodegeneration in the hippocampus [6–10]. We have previously shown that A*β*_25–35_, like A*β*_1–42_, exerted neurotoxic effects on SH-SY5Y cells [11]. These data are among numerous studies confirming that A*β*_25–35_ is a useful tool for investigating AD-related mechanisms in animal models [10].

Oxidative stress has been strongly implicated in the pathophysiology of AD [12]. Increased free radicals can damage proteins, lipids, and nucleic acids. The combination of mitochondrial dysfunction and A*β* accumulation generates reactive oxygen species (ROS) which, in the presence of metal ions such as Fe^2+^ and Cu^2+^, may contribute to oxidative damage in AD brains [13]. In addition, dysregulated brain iron homeostasis also accelerates AD progression. Excessive iron in the brain can directly lead to the generation of free radicals that eventually cause neurodegenerative disease.

Mitochondrial ferritin (FtMt) is a recently identified ferritin that accumulates specifically in the mitochondria and possesses a high homology to H-ferritin [14]. The functions of FtMt include iron storage, regulating iron distribution between the cytosol and mitochondrial compartments, and preventing the production of ROS generated through the Fenton reaction [15, 16]. FtMt has been reported to be present in relatively low abundance in the liver and splenocytes, the major iron storage sites, while FtMt is found at higher levels in the testis, kidney, heart and brain, and tissues with high metabolic activity. Together with the knowledge that H-ferritin expression confers an antioxidant effect, the tissue distribution of FtMt is in line with a protective function of FtMt in mitochondria against iron-dependent oxidative damage [17]. Previous results indicated that FtMt was involved in the pathogenesis of neurodegenerative diseases, including AD, Parkinson's disease, and Friedreich's ataxia [18–20]. Increased expression of FtMt has been observed in the brains of AD patients and is associated with the antioxidant role of this protein [2]. Furthermore, our previous studies have shown that FtMt exerted a neuroprotective effect against 6-hydroxydopamine- (6-OHDA-) induced dopaminergic cell damage [20] and FtMt overexpression attenuated A*β*-induced neurotoxicity [11].

Although abnormal iron metabolism and oxidative stress have been reported in AD, little information is available about the role of FtMt in the pathogenesis of AD. In the present study, we investigated memory impairment and neuronal cell death in A*β*_25–35_-injected* Ftmt* knockout mice. In addition, we explored the molecular mechanisms responsible for neuronal damage in this model. Our data indicate that FtMt deficiency exacerbated A*β*_25–35_-induced neuronal cell damage by altering intracellular iron levels in a way that intensifies the oxidative stress caused by A*β*_25–35_.

## 2. Materials and Methods

### 2.1. Animals

C57BL/6* Ftmt*-null mice were obtained from The Jackson Laboratory [21]. Mice were housed under conditions controlled for temperature (22°C) and humidity (40%), using a 12 hr/12 hr light/dark cycle [22]. Mice were fed a standard rodent diet and water ad libitum. Age-matched C57BL/6J wild-type male mice and* Ftmt* knockout male mice (10 months) were used in this study. All procedures were carried out in accordance with the National Institutes of Health Guide for the Care and Use of Laboratory Animals and were approved by the Animal Care and Use Committee of the Hebei Science and Technical Bureau in China.

### 2.2. Antibodies and Reagents

The following antibodies and reagents were used: *β*-actin (Alpha Diagnostic International, USA), TfR1 (Sigma-Aldrich, USA), FPN1, DMT1 (+IRE) and DMT1 (−IRE) (Alpha Diagnostic International, USA), L-ferritin (Abcam Inc., SF, USA), cleaved PARP, caspase-3, phospho-p38 (p-p38) and p38 (Cell Signaling Technology, USA), Bcl-2 and Bax (Santa Cruz Biotechnology, USA), A*β*_25–35_ peptide (Sigma-Aldrich, USA), and TUNEL in situ Cell Death Detection Kit (Roche Diagnostics GmbH, Mannheim, Germany).

### 2.3. Drug Preparation and Injection

A*β*_25–35_ was dissolved in sterile saline and aggregated by incubation at 37°C for 4 days before use [23]. The aggregated form of A*β*_25–35_ (7.5 nmol in 5 *μ*L saline per injection) was injected into the right lateral ventricle as previously described [24]. Mice were randomly divided into four groups: wild-type with saline injection (WT + saline), wild-type with A*β*_25–35_ injection (WT + A*β*_25–35_),* Ftmt* knockout with saline injection (KO + saline), and* Ftmt* knockout with A*β*_25–35_ injection (KO + A*β*_25–35_). After injection, the mice were housed for 15 days under normal conditions and then trained and tested in a Morris water maze (MWM) as described below.

### 2.4. Morris Water Maze Test (MWM Test)

Spatial learning and memory deficits were assessed using the Morris water maze as described previously [25, 26], with minor modification. The experimental apparatus consisted of a circular tank (diameter = 120 cm, height = 50 cm) that was divided into four quadrants, filled with water, and maintained at 22 ± 2°C. At first, a visible platform test was performed, which confirmed that there were no significant differences in sensory, motor, or motivational activities among these four groups. Then, hidden platform tests were performed in succession. For the hidden platform test, a round platform (diameter = 9 cm) was placed at the midpoint of the fourth quadrant, 1 cm below the water surface. The test was conducted four times a day for four days, with four randomized starting points. The position of the escape platform was kept constant. Each trial lasted for 90 s or ended as soon as the mice reached the submerged platform.

### 2.5. Probe Test

To assess memory consolidation, a probe test was performed 24 h after the Morris water maze test [25]. For the probe test, the platform was removed and the mice were allowed to swim freely. The swimming pattern of every mouse was recorded for 90 s with a camera. Consolidated spatial memory was estimated by the time spent in the target quadrant area.

### 2.6. Assessment of Apoptosis

After the behavioral testing, the animals were perfused with 0.9% saline under anesthesia with 0.4% Nembutal. The brains were immediately collected and then postfixed with 4% paraformaldehyde in 0.1 M phosphate buffer. Serial coronal sections were cut at 15 *μ*m on a freezing microtome (Leica CM1950, Leica Microsystems, Shanghai, China) and mounted onto slides covered with APES (Beijing Zhongshan Biotechnology, Beijing, China). The presence of apoptosis in the dentate gyrus of mouse hippocampi was assessed by the terminal deoxynucleotidyl transferase-mediated FITC-dUTP nick-end labeling method (TUNEL) following the manufacturer's protocol. Nuclei were counterstained with DAPI. The number of TUNEL-DAPI-positive cells was counted as described previously [27]. The counting area was located in the same position in all groups. For each group, quantification was performed in sections from three different mice.

### 2.7. Western Blot Analysis

Protein expression was assessed by western blotting as previously described [28], with minor modifications. Briefly, hippocampi were homogenized and sonicated in RIPA buffer containing 1% NP40 and protease inhibitor cocktail tablets (Roche Diagnostics GmbH, Roche Applied Science, 68298 Mannheim, Germany). After centrifugation at 12,000 ×g for 20 min at 4°C, the supernatant was collected, and the whole cell lysate protein concentration was measured using the BCA Protein Quantification Kit (Yeasen Biotechnology, Shanghai, China). Protein from each sample (40 mg) was resolved by SDS-PAGE on 12% or 10% gels and then transferred to PVDF membranes. The blots were blocked in 5% nonfat milk containing 20 mM Tris-HCl (pH 7.6, 137 mM NaCl, and 0.1% Tween-20; TBS-T) for 1.5 h at room temperature, followed by incubation with primary antibody overnight at 4°C. After washing three times with TBS-T, the blots were incubated with horseradish peroxide (HRP) conjugated secondary antibody for 1.5 h at room temperature. Immunoreactive proteins were detected using the enhanced chemiluminescence (ECL) method and quantified by transmittance densitometry using volume integration with Multi Gauge ver. 3.1 software (FUJIFILM Corporation, Tokyo, Japan).

### 2.8. Measurement of MDA and SOD

Malondialdehyde (MDA), a marker of lipid peroxidation, was assessed using the thiobarbituric acid (TBA) method [29] using a kit from the Nanjing Jiancheng Bioengineering Institute (Nanjing, China) according to the manufacturer's instructions. This method is based on the spectrophotometric measurement of the product of the reaction of TBA with MDA. MDA concentrations were then calculated by the absorbance of TBA reactive substances (TBARS) at 532 nm.

Superoxide dismutases (SODs), which catalyze the dismutation of superoxide into oxygen and hydrogen peroxide, were determined according to xanthine oxidase method using a commercial kit (Nanjing Jiancheng Bioengineering Institute, Nanjing, China) according to the manufacturer's instructions. The xanthine-xanthine oxidase system produces superoxide ions, which can react with 2-(4-iodophenyl)-3-(4-nitrophenol-5-phenlyltetrazolium chloride) to form a red formazan dye, which can be detected by its absorbance at 550 nm [29].

The levels of MDA and the total SOD (T-SOD) activity were determined in each group. The hippocampi of mice were homogenized in ice-cold saline. The homogenate was centrifuged at 3000 ×g at 4°C for 15 min, and the supernatant was used to determine T-SOD activity and MDA levels with a spectrophotometer (Synergy H4, BioTek, USA) at wavelengths of 550 nm and 532 nm, respectively. Each group contained five mice for the MDA and SOD tests, with each test repeated three times.

### 2.9. Statistical Analysis

All data are expressed as the mean ± standard deviation. One-way analysis of variance was used to estimate overall significance and was followed by Tukey's post hoc test corrected for multiple comparisons. A probability level of 95% (*p* < 0.05) was considered significant. All the tests were performed with SPSS 21.0 (IBM SPSS21.0, Armonk, New York, United States).

## 3. Results

### 3.1. *Ftmt* Ablation Exacerbates A*β*_25–35_-Induced Spatial Memory Deficits

**Figure 1:**
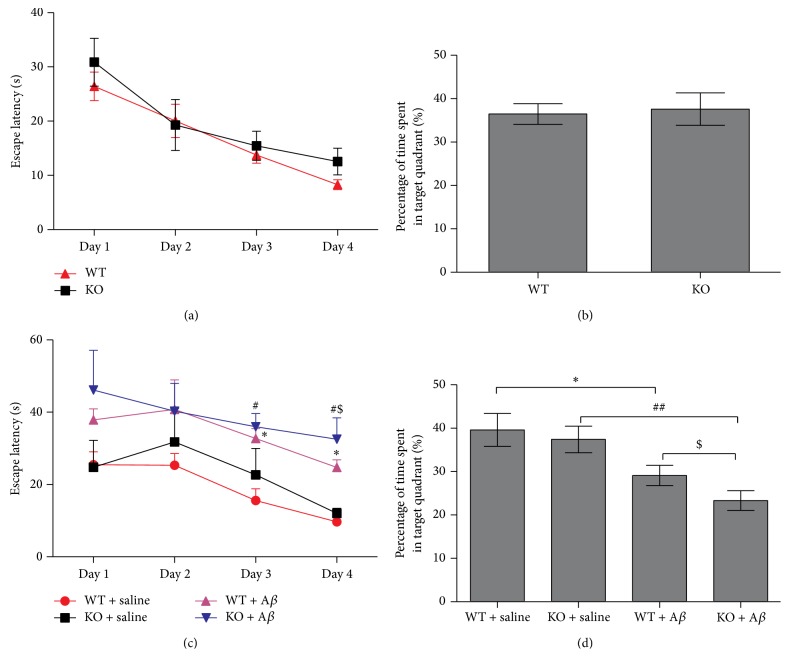
The effect of* Ftmt* ablation on A*β*_25–35_-induced spatial memory deficits. (a) Age-matched (10 months old)* Ftmt* knockout mice (*n* = 20) and wild-type mice (*n* = 23) were administered a 90 s trial four times a day to find the hidden platform. The analysis of the recorded data shows the changes in latency to find the hidden platform over the four consecutive days of training. (b)* Ftmt* knockout mice and wild-type mice were assessed in the probe test one day after the hidden platform test. The time spent in the target quadrant within the 90 s was recorded. (c) The effect of A*β*_25–35_ on escape latency. (Wild-type mice and* Ftmt* knockout mice were randomly divided into four groups and injected with A*β*_25–35_ or saline. Fifteen days later, the MWM test was conducted.) (d) The time spent in the target quadrant during the probe test after injecting A*β*_25–35_. The data is presented as the mean ± SD. ^*∗*^*p* < 0.05 versus WT + saline group, *n* = 11. ^#^*p* < 0.05, ^##^*p* < 0.01 versus KO + saline group, *n* = 10. ^$^*p* < 0.05 versus WT + A*β*_25–35_ group, *n* = 10. KO + A*β*_25–35_, *n* = 10.

The MWM test was conducted to assess learning and memory in 10-month-old wild-type mice (WT) and* Ftmt* knockout mice (KO). All mice were trained with four trials per day for 4 days. “Escape latency” is the time to reach the platform in the water maze and is used as a proxy for mouse memory. Compared to wild-type mice,* Ftmt* knockout mice took approximately the same time to reach the platform after training (Figure 1(a)). After the water maze test, we performed a probe test using the metric “time spent in quadrant” to investigate the maintenance of memory. The time spent in the target quadrant was also similar in wild-type and* Ftmt* knockout mice (Figure 1(b)). After treatment with A*β*_25–35_, both the WT + A*β*_25–35_ group and the KO + A*β*_25–35_ group took a significantly longer time to reach the platform than the groups without A*β*_25–35_ injection (Figure 1(c)). Furthermore, A*β*_25–35_-infused* Ftmt* knockout mice had a significantly greater memory impairment (longer escape latency time) than A*β*_25–35_-infused wild-type mice. In addition, the time spent in the target quadrant in the probe trial was less in both the WT + A*β*_25–35_ and the KO + A*β*_25–35_ groups than in the control groups. Importantly, the KO + A*β*_25–35_ group was in the target quadrant for even less time than the WT + A*β*_25–35_ group (Figure 1(d)). Overall, our results show that knockout of* Ftmt* in mice significantly exacerbates memory deficits in the A*β*_25–35_-induced AD model.

### 3.2. *Ftmt* Ablation Enhances A*β*_25–35_-Induced Neuronal Cell Apoptosis

**Figure 2:**
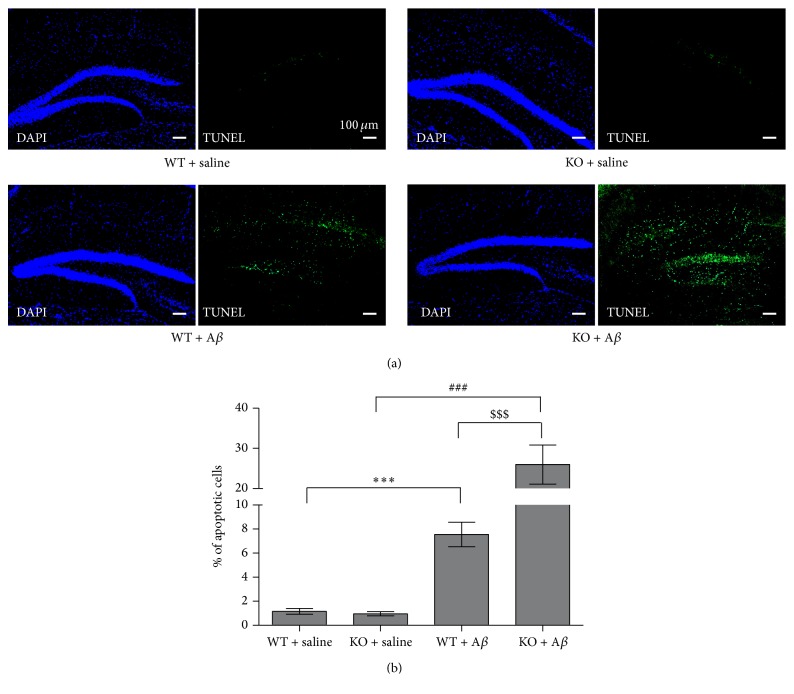
The effect of* Ftmt* ablation on A*β*_25–35_ -induced neuronal cell apoptosis. Apoptotic cell death was assessed by DAPI and TUNEL staining, as described in the Materials and Methods section. (a) Representative photographs (original magnification 100x) of the dentate gyrus of the hippocampus of mouse brains. (b) The statistical analysis of relative apoptotic cell levels. Data are presented as the mean ± SD, *n* = 3. ^*∗∗∗*^*p* < 0.001 versus WT + saline group, ^###^*p* < 0.001 versus KO + saline group, and ^$$$^*p* < 0.001 versus WT + A*β*_25–35_ group.

To evaluate the neuronal apoptosis affected by* Ftmt* gene ablation in the AD model, we used the TUNEL method to detect apoptosis after A*β*_25–35_ stimulation. Our results indicated that neuronal apoptosis in the hippocampi was increased after injecting A*β*_25–35_, especially in the dentate gyrus. The number of apoptotic cells in the WT + A*β*_25–35_ group was approximately four times greater than that observed in the WT + saline group, and there was also a noticeable increase in the KO group. The number of apoptotic cells in the KO + A*β*_25–35_ group was more than threefold that of the WT + A*β*_25–35_ group. These results confirmed that* Ftmt* knockout significantly enhanced neuronal apoptosis compared to the WT + A*β*_25–35_ group (Figures 2(a) and 2(b)), suggesting that FtMt is protective against A*β*_25–35_-induced apoptosis.

### 3.3. FtMt Deficiency in the AD Mouse Model Elevates Proapoptotic Signals

**Figure 3:**
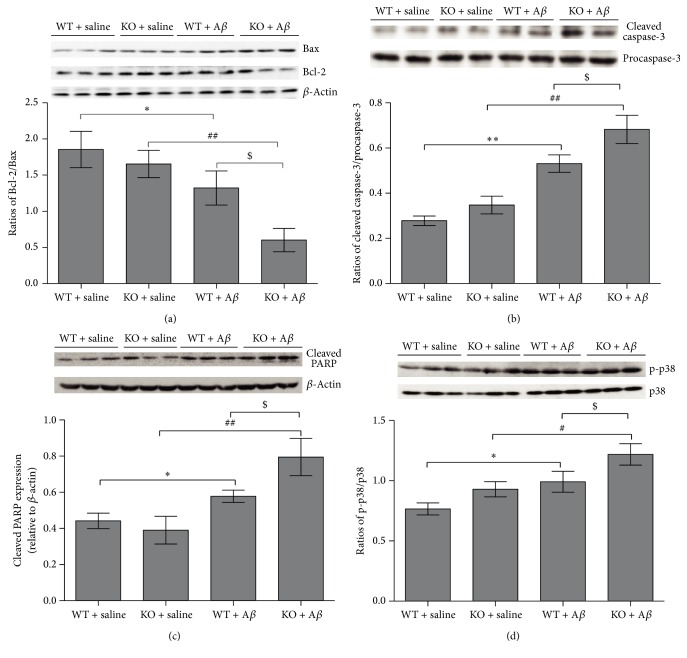
The effect of* Ftmt* deficiency on the Bcl-2/Bax ratio, cleaved caspase-3, and p38 MAPK activation in mice. Western blot and subsequent densitometric analysis of (a) the ratio of Bcl-2/Bax, (b) the amount of cleaved caspase-3, (c) the amount of cleaved PARP, and (d) the ratio of p-p38/p38. Data are presented as the mean ± SD, *n* = 3. ^*∗*^*p* < 0.05, ^*∗∗*^*p* < 0.01 versus WT + saline group, ^#^*p* < 0.05, ^##^*p* < 0.01 versus KO + saline group, and ^$^*p* < 0.05 versus WT + A*β*_25–35_ group. MAPK: mitogen-activated protein kinase.

We found that the knockout of* Ftmt* remarkably decreased the ratio of Bcl-2/Bax (Figure 3(a)) and increased the activation of cleaved caspase-3 (Figure 3(b)) after A*β*_25–35_ treatment in mice. In the apoptotic cascade, caspase-3 cleaves poly-ADP-ribose-polymerase (PARP), leading to the accumulation of an 89 kDa PARP fragment [30]. Caspase-3-mediated PARP cleavage was enhanced in the KO + A*β*_25–35_ group compared to the WT + A*β*_25–35_ group (Figure 3(c)). These results indicate that the lack of FtMt can affect the Bcl-2/Bax ratio, leading to caspase-3 activation and a concomitant increase in PARP cleavage and, ultimately, apoptosis after A*β*_25–35_ injection.

The activation of p38 (MAP kinase) by phosphorylation is implicated in oxidative stress-induced cell death [31]. A high p-p38/p38 ratio can simultaneously promote Bax expression and decrease Bcl-2 levels. A*β*_25–35_ significantly induced the activation of p38 in the hippocampus. In the KO + A*β*_25–35_ group, p-p38 levels were elevated (Figure 3(d)). Overall, our data demonstrate that the knockout of* Ftmt* in mice injected with A*β*_25–35_ increases p-p38 levels which alters the amounts of proteins related to cell death, ultimately leading to increased neuronal cell death in the hippocampus.

### 3.4. *Ftmt* Knockout Increases MDA Levels in AD Mice without Altering SOD

**Figure 4:**
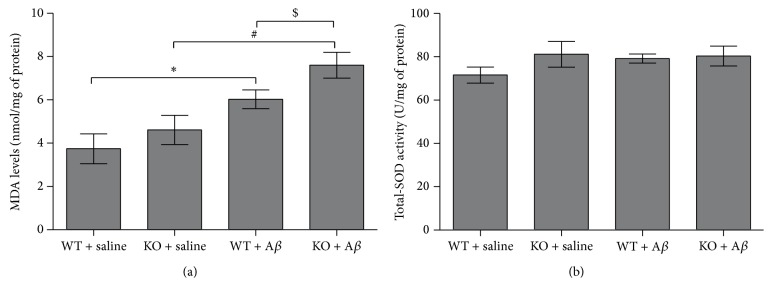
The effects of* Ftmt* ablation on the levels of MDA and Total SOD. (a) MDA and (b) total SOD were assayed as described in the Materials and Methods section. Values are presented as the mean ± SD. ^*∗*^*p* < 0.05 versus WT + saline group, ^#^*p* < 0.05 versus KO + saline group, and ^$^*p* < 0.05 versus WT + A*β*_25–35_ group.

To determine whether increased levels of oxidative stress are responsible for the increased apoptosis in the hippocampus in the AD mouse model, we examined the levels of MDA and the activity of SOD in each group. Free radicals attack polyunsaturated fatty acids, leading to structural damage to membranes and the generation of MDA, which is considered a marker of lipid peroxidation and thus a surrogate for oxidative damage [32]. The level of MDA was increased in AD mice compared with controls, but this increase was significantly greater in* Ftmt* knockout mice (Figure 4(a)). SOD is a free radical scavenging enzyme that converts superoxide into H_2_O_2_. The content of total SOD was unchanged in the four groups (Figure 4(b)).

### 3.5. The Effects of* Ftmt* Knockout on the Levels of L-Ferritin, TfR1, DMT1, and FPN1

**Figure 5:**
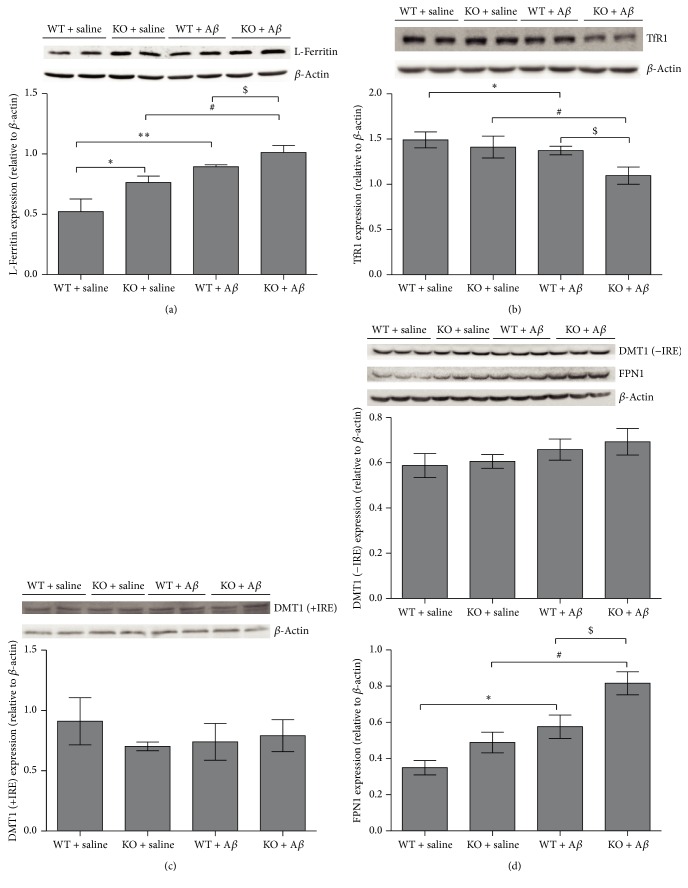
The effects of* Ftmt* deficiency on the levels of L-ferritin, TfR1, DMT1, and FPN1. Western blotting was used to assay iron metabolism related proteins in hippocampus of mice. (a) L-ferritin. (b) TfR1. (c) DMT1 (+IRE). (d) FPN1 and DMT1-IRE. The expression levels of these proteins were normalized to *β*-actin and expressed as the mean ± SD. ^*∗*^*p* < 0.05, ^*∗∗*^*p* < 0.01 versus WT + saline group, ^#^*p* < 0.05 versus KO + saline group, and ^$^*p* < 0.05 versus WT+ A*β*_25–35_ group.

Iron is an essential cofactor in many proteins, but excess free iron contributes to enhanced generation of ROS and oxidative stress [33]. When treated with A*β*_25–35_, the levels of L-ferritin were upregulated while those of TfR1 decreased significantly, compared to the control groups. The highest amount of L-ferritin expression was observed in the KO + A*β*_25–35_ group (Figures 5(a) and 5(b)). In addition, the content of L-ferritin was also increased in the KO + saline group when compared to the WT + Saline group (Figure 5(a)). These observations indicated that A*β*_25–35_ stimulation may lead to alterations in iron homeostasis and that FtMt deficiency may accelerate this process. In addition, alterations in cellular iron distribution (as detected by Perls' staining) (see Supplementary Figure  1 of the Supplementary Material available online at https://doi.org/10.1155/2017/1020357) support this hypothesis. However, there was no significant difference in the expression of DMT1 (+IRE) or DMT1 (−IRE) in any group (Figures 5(c) and 5(d)), while the expression of FPN1, the iron release protein, was increased in both groups treated with A*β*_25–35_ (Figure 5(d)). These results suggest that injection of A*β*_25–35_ into the brain disturbed iron homeostasis, possibly leading to oxidative damage, both of which were exacerbated by the lack of FtMt.

## 4. Discussion

Iron is an essential trace element for human health. The metal participates in many biological processes. Iron homeostasis is stringently regulated in vivo as excess iron can catalyze the generation of oxidative damage [20]. Importantly, iron is considered a contributing neurotoxic factor in several neurodegenerative disorders, including AD [34]. Cortical iron elevation has been increasingly reported as a feature of AD [35] and may contribute to the oxidative damage observed in AD brains. In addition, abnormalities in iron-regulatory proteins occur in the brains of AD sufferers [19]. FtMt, a recently identified H-ferritin-like protein expressed only in mitochondria, is thought to function to protect mitochondria from iron-dependent oxidative damage in cells with high metabolic activity and oxygen consumption. Previous studies have already shown an increased FtMt expression in the hippocampus of AD patients [36]. In addition, the downregulation of FtMt causes severe neurodegeneration in the Purkinje cells of the cerebellum [20]. In this study,* Ftmt* gene knockout mice were firstly used to study the effects of FtMt on the behavioral changes and mechanisms of A*β*_25–35_-induced neurotoxicity.

Previous results indicate that FtMt-deficient mice are healthy and do not show any evident phenotype under baseline feeding conditions [21]. Here we have also found that 10-month-old, wild-type, and* Ftmt* knockout mice show no behavior or memory differences, as determined by MWM assays. Thus, FtMt deficiency has no obvious effects in the mouse brain under normal physiological conditions. To further elucidate the role of FtMt in AD pathogenesis, we first showed that intracerebroventricular infusion of A*β*_25–35_ exacerbates memory impairment in* Ftmt* knockout mice compared to the A*β*_25–35_-infused controls. The number of apoptotic cells in the hippocampus was also significantly increased in A*β*_25–35_-infused* Ftmt* knockout mice, which may account for their poorer performance in the MWM. Our data suggest that FtMt is not essential in mice under normal conditions. However, when challenged, such as with amyloid beta treatment, there appears to be a need for FtMt in a neuroprotective role.

Bcl-2 and Bax play important roles in oxidative stress-mediated neuronal apoptosis [37]. It has been reported that Bcl-2 protects neurons against oxidant stress and apoptosis in PD [38]. Bcl-2 also maintains mitochondrial integrity by blocking the release of apoptotic factors from mitochondria into cytoplasm [20]. Bax can promote cell death by activating elements of the caspase pathway [39], especially caspase-3 [40]. As previously described, the activation of caspases, a family of cysteine proteases, is a central mechanism in the apoptotic process. Our results show that knockout of* Ftmt* decreases the ratio of Bcl-2/Bax and increases the activation of caspase-3 and PARP cleavage, which ultimately leads to cell death.

Accumulated evidence demonstrated that A*β*-induced neuronal injury triggers transcriptional and posttranscriptional processes that regulate neuronal fate, including the activation of the MAPK pathway [41]. In this signaling cascade, p38 MAPK is activated by phosphorylation, and a high p-p38/p38 ratio can simultaneously promote Bax expression and decrease Bcl-2 levels. Our results show a significant elevation of p-p38 levels and its downstream factor Bax, strongly suggesting that this apoptotic signal transduction pathway is enhanced in* Ftmt* knockout mice treated with A*β*_25–35_.

An increasing number of studies have suggested that oxidative stress is associated with AD neurodegeneration and caspase-mediated apoptosis [42]. We detected a marked increase in the level of MDA, an indicator of oxidative damage, in the hippocampi of* Ftmt* knockout mice, indicating that knockout of* Ftmt* aggravates oxidative stress. Previous studies indicate that, in certain antioxidant systems, there might be a time lag between the synthesis of protein and the expression of mRNA following neurotoxicity; the activity of SOD is altered in the process of A*β*_25–35_ induced injury. Cu, Zn-SOD, and Mn-SOD activity in hippocampi of A*β*_1–42_ treated mice returned to near vehicle levels after 10 days [43]. Our data show that* Ftmt* ablation did not significantly affect the activity of total SOD, although this may be related to the time point at which SOD activity was measured.

Cellular iron homeostasis is maintained by a strict regulation of various proteins that are involved in iron uptake, export, storage, and utilization [44]. Studies from our group and others have demonstrated that aberrant iron homeostasis can generate ROS which can eventuate in AD pathogenesis [2, 11]. In the present study, we observed upregulated L-ferritin and FPN1 and a simultaneous decrease in TfR1. These changes are likely to be the result of inhibited iron-regulatory protein binding [45] brought about by an increase in the regulatory iron pool in the neuronal cells injected with A*β*. Consistent with this, the absence of FtMt may decrease the cells ability to sequester excess iron under stressed conditions and enhances the degree of changes in the measured proteins of iron metabolism. Our previous data also indicate that “uncommitted” iron levels, commonly referred to as the “labile iron pool” (LIP), significantly increased in SH-SY5Y cells treated with A*β*_25–35_. FtMt overexpression was able to reverse this change [11]. We propose that a larger LIP, resulting from a redistribution of iron from mitochondria to the cytosol, especially in the absence of FtMt, is responsible for the oxidative stress that mediates the damage of cell components in our AD model [46].

**Figure 6:**
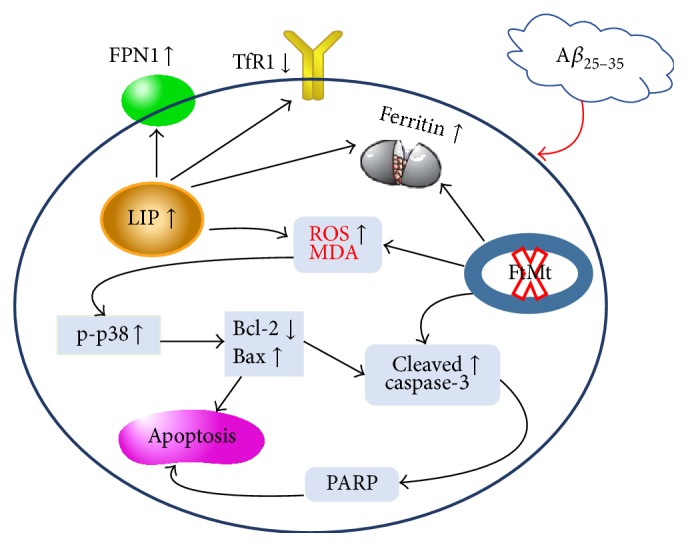
A schematic representation of the mechanism leading to neuronal cell apoptosis induced by A*β*_25–35_ in mice with a disrupted* Ftmt* gene. A*β*_25–35_ changes the levels (LIP) and distribution of intracellular iron, thus increasing oxidative stress. Without* Ftmt* to sequester excess mitochondrial iron, lipid peroxidation and the level of LIP are significantly increased. These changes may signal the cell to begin the process of programmed death through the P38 MAPK pathway, resulting in neuronal cell death, which is enhanced in* Ftmt* knockout mice, leading to worsened memory impairments.

In summary, our research indicates that A*β*_25–35_ elevates the LIP and causes oxidative stress, which can be exacerbated by the lack of FtMt. The excess iron donates electrons to generate ROS and lipid peroxidation. These changes initiate the programmed cell death through the p38/MAPK pathway, ultimately causing neuronal apoptosis which causes more severe memory impairment. The alteration of iron maybe provides the feedback regulation to the levels of TfR1 and FPN1 (Figure 6).

## 5. Conclusion

The current study supports the hypothesis that the functionality of FtMt is not essential under normal conditions. But, in cases of neuronal stress, such as A*β*_25–35_ accumulation, FtMt offers a profound neuroprotection through regulating cellular iron content and distribution in a way that keeps oxidative stress in check, preventing the activation of apoptosis.

## Supplementary Material

Serial sections (15 μm) were cut using a cryostat microtome (Leica CM1950) and mounted on slides. Prior to staining, sections were treated with methanol containing 3% hydrogen peroxide for 10 minutes. After washing with 0.01 M PBS, sections were stained with Prussian blue, mixing 1% potassium ferrocyanide with 1% HCl for 8 hours at 37°C. Then sections were washed in 0.01 M PBS and treated with DAB solution for 5 minutes. PBS, phosphate buffered saline.

## References

[1] Campbell V. A., Gowran A. (2007). Alzheimer's disease; taking the edge off with cannabinoids?. *British Journal of Pharmacology*.

[2] Yang H., Guan H., Yang M. (2015). Upregulation of mitochondrial ferritin by proinflammatory cytokines: implications for a role in Alzheimer's disease. *Journal of Alzheimer's Disease*.

[3] Selkoe D. J. (2001). Alzheimer's disease: genes, proteins, and therapy. *Physiological Reviews*.

[4] Stéphan A., Phillips A. G. (2005). A case for a non-transgenic animal model of Alzheimer's disease. *Genes, Brain and Behavior*.

[5] Yankner B. A., Duffy L. K., Kirschner D. A. (1990). Neurotrophic and neurotoxic effects of amyloid *β* protein: reversal by tachykinin neuropeptides. *Science*.

[6] Maurice T., Lockhart B. P., Privat A. (1996). Amnesia induced in mice by centrally administered *β*-amyloid peptides involves cholinergic dysfunction. *Brain Research*.

[7] Stepanichev M. Y., Moiseeva Y. V., Lazareva N. A., Onufriev M. V., Gulyaeva N. V. (2003). Single intracerebroventricular administration of amyloid-beta (25–35) peptide induces impairment in short-term rather than long-term memory in rats. *Brain Research Bulletin*.

[8] Stepanichev M. Y., Zdobnova I. M., Zarubenko I. I. (2004). Amyloid-*β*(25–35)-induced memory impairments correlate with cell loss in rat hippocampus. *Physiology and Behavior*.

[9] Yamaguchi Y., Kawashima S. (2001). Effects of amyloid-*β*-(25–35) on passive avoidance, radial-arm maze learning and choline acetyltransferase activity in the rat. *European Journal of Pharmacology*.

[10] Stepanichev M. Y., Onufriev M. V., Yakovlev A. A. (2008). Amyloid-*β* (25-35) increases activity of neuronal NO-synthase in rat brain. *Neurochemistry International*.

[11] Wu W. S., Zhao Y. S., Shi Z. H. (2013). Mitochondrial ferritin attenuates *β*-amyloid-induced neurotoxicity: reduction in oxidative damage through the Erk/P38 mitogen-activated protein kinase pathways. *Antioxidants & Redox Signaling*.

[12] Darvesh A. S., Carroll R. T., Bishayee A., Geldenhuys W. J., Van Der Schyf C. J. (2010). Oxidative stress and Alzheimer's disease: dietary polyphenols as potential therapeutic agents. *Expert Review of Neurotherapeutics*.

[13] Choi D.-Y., Lee Y.-J., Hong J. T., Lee H.-J. (2012). Antioxidant properties of natural polyphenols and their therapeutic potentials for Alzheimer's disease. *Brain Research Bulletin*.

[14] Levi S., Corsi B., Bosisio M. (2001). A human mitochondrial ferritin encoded by an intronless gene. *Journal of Biological Chemistry*.

[15] Corsi B., Cozzi A., Arosio P. (2002). Human mitochondrial ferritin expressed in HeLa cells incorporates iron and affects cellular iron metabolism. *Journal of Biological Chemistry*.

[16] Drysdale J., Arosio P., Invernizzi R. (2002). Mitochondrial ferritin: a new player in iron metabolism. *Blood cells, molecules & diseases*.

[17] Santambrogio P., Biasiotto G., Sanvito F., Olivieri S., Arosio P., Levi S. (2007). Mitochondrial ferritin expression in adult mouse tissues. *Journal of Histochemistry and Cytochemistry*.

[18] Gao G., Chang Y.-Z. (2014). Mitochondrial ferritin in the regulation of brain iron homeostasis and neurodegenerative diseases. *Frontiers in Pharmacology*.

[19] Yang H., Yang M., Guan H. (2013). Mitochondrial ferritin in neurodegenerative diseases. *Neuroscience Research*.

[20] Shi Z.-H., Nie G., Duan X.-L. (2010). Neuroprotective mechanism of mitochondrial ferritin on 6-hydroxydopamine- induced dopaminergic cell damage: implication for neuroprotection in parkinson's disease. *Antioxidants and Redox Signaling*.

[21] Bartnikas T. B., Campagna D. R., Antiochos B., Mulhern H., Pondarré C., Fleming M. D. (2010). Characterization of mitochondrial ferritin-deficient mice. *American Journal of Hematology*.

[22] You L.-H., Li F., Wang L. (2015). Brain iron accumulation exacerbates the pathogenesis of MPTP-induced Parkinson's disease. *Neuroscience*.

[23] Mamiya T., Asanuma T., Kise M. (2004). Effects of pre-germinated brown rice on beta-amyloid protein-induced learning and memory deficits in mice. *Biological and Pharmaceutical Bulletin*.

[24] Park S. H., Kim J. H., Bae S. S. (2011). Protective effect of the phosphodiesterase III inhibitor cilostazol on amyloid *β*-induced cognitive deficits associated with decreased amyloid *β* accumulation. *Biochemical and Biophysical Research Communications*.

[25] Chen C., Li X., Gao P. (2015). Baicalin attenuates Alzheimer-like pathological changes and memory deficits induced by amyloid *β*_1–42_ protein. *Metabolic Brain Disease*.

[26] Takeda S., Sato N., Niisato K. (2009). Validation of A*β*1-40 administration into mouse cerebroventricles as an animal model for Alzheimer disease. *Brain Research*.

[27] Huang W., Xie W.-B., Qiao D. (2015). Caspase-11 plays an essential role in methamphetamine-induced dopaminergic neuron apoptosis. *Toxicological Sciences*.

[28] You L.-H., Li Z., Duan X.-L., Zhao B.-L., Chang Y.-Z., Shi Z.-H. (2016). Mitochondrial ferritin suppresses MPTP-induced cell damage by regulating iron metabolism and attenuating oxidative stress. *Brain Research*.

[29] Mao G.-X., Zheng L.-D., Cao Y.-B. (2012). Antiaging effect of pine pollen in human diploid fibroblasts and in a mouse model induced by D-galactose. *Oxidative Medicine and Cellular Longevity*.

[30] Nicholson D. W., Ali A., Thornberry N. A. (1995). Identification and inhibition of the ICE/CED-3 protease necessary for mammalian apoptosis. *Nature*.

[31] Wang X., Martindale J. L., Liu Y., Holbrook N. J. (1998). The cellular response to oxidative stress: influences of mitogen-activated protein kinase signalling pathways on cell survival. *Biochemical Journal*.

[32] Lovell M. A., Markesbery W. R. (2007). Oxidative damage in mild cognitive impairment and early Alzheimer's disease. *Journal of Neuroscience Research*.

[33] Ward R. J., Zucca F. A., Duyn J. H., Crichton R. R., Zecca L. (2014). The role of iron in brain ageing and neurodegenerative disorders. *The Lancet Neurology*.

[34] Mancuso C., Scapagnini G., Currò D. (2007). Mitochondrial dysfunction, free radical generation and cellular stress response in neurodegenerative disorders. *Frontiers in Bioscience*.

[35] van Rooden S., Doan N. T., Versluis M. J. (2015). 7T T2∗-weighted magnetic resonance imaging reveals cortical phase differences between early- and late-onset Alzheimer's disease. *Neurobiology of Aging*.

[36] Wang L., Yang H., Zhao S. (2011). Expression and localization of mitochondrial ferritin mRNA in Alzheimer's disease cerebral cortex. *PLoS ONE*.

[37] Oshima Y., Akiyama T., Hikita A. (2008). Pivotal role of Bcl-2 family proteins in the regulation of chondrocyte apoptosis. *Journal of Biological Chemistry*.

[38] Tortosa A., López E., Ferrer I. (1997). Bcl-2 and Bax proteins in lewy bodies from patients with Parkinson's disease and Diffuse Lewy body disease. *Neuroscience Letters*.

[39] Golstein P. (1997). Controlling cell death. *Science*.

[40] Zheng T. S., Hunot S., Kuida K. (2000). Deficiency in caspase-9 or caspase-3 induces compensatory caspase activation. *Nature Medicine*.

[41] Yang Z. H., Sun K., Suo W. H. (2010). N-stearoyltyrosine protects primary neurons from A*β*-induced apoptosis through modulating mitogen-activated protein kinase activity. *Neuroscience*.

[42] Kruman I. I., Nath A., Mattson M. P. (1998). HIV-1 protein tat induces apoptosis of hippocampal neurons by a mechanism involving caspase activation, calcium overload, and oxidative stress. *Experimental Neurology*.

[43] Jhoo J. H., Kim H.-C., Nabeshima T. (2004). *β*-Amyloid (1-42)-induced learning and memory deficits in mice: involvement of oxidative burdens in the hippocampus and cerebral cortex. *Behavioural Brain Research*.

[44] Santamaria R., Fiorito F., Irace C. (2011). 2,3,7,8-Tetrachlorodibenzo-p-dioxin impairs iron homeostasis by modulating iron-related proteins expression and increasing the labile iron pool in mammalian cells. *Biochimica et Biophysica Acta—Molecular Cell Research*.

[45] Lane D. J. R., Merlot A. M., Huang M. L.-H. (2015). Cellular iron uptake, trafficking and metabolism: key molecules and mechanisms and their roles in disease. *Biochimica et Biophysica Acta—Molecular Cell Research*.

[46] Valko M., Jomova K., Rhodes C. J., Kuča K., Musílek K. (2016). Redox- and non-redox-metal-induced formation of free radicals and their role in human disease. *Archives of Toxicology*.

