# Supplementary material for: Mitochondrial Ferritin Deletion Exacerbates β-Amyloid-Induced Neurotoxicity in Mice
Source: Oxid Med Cell Longev. 2017 Jan 16;2017:1020357. doi: 10.1155/2017/1020357 (PMC5278219; doi:10.1155/2017/1020357)
Supplement: Supplementary file 1 — Serial sections (15 μm) were cut using a cryostat microtome (Leica CM1950) and mounted on slides. Prior to staining, sections were treated with methanol containing 3% hydrogen peroxide for 10 minutes. After washing with 0.01 M PBS, sections were stained with Prussian blue, mixing 1% potassium ferrocyanide with 1% HCl for 8 hours at 37°C. Then sections were washed in 0.01 M PBS and treated with DAB solution for 5 minutes. PBS, phosphate buffered saline. [file 1020357.f1.pdf]

Supplementary Information

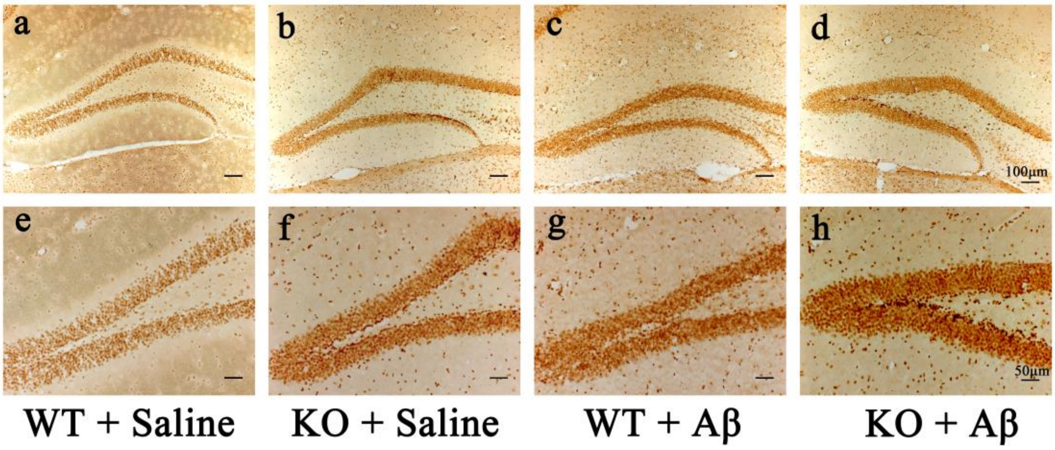

Supplementary Fig. 1 **The effects of *Ftmt* deficiency on iron accumulation in hippocampus.** To confirm the effect of FtMt on iron metabolism after $A\beta_{25-35}$ injection, we detected iron levels of hippocampi in each group, using Perls'+DAB (3,3′-diaminobenzidine) method [1-4]. Serial sections (15μm) were cut using a cryostat microtome (Leica CM1950) and mounted on slides. Prior to staining, sections were treated with methanol containing 3% hydrogen peroxide for 10 minutes. After washing with 0.01M PBS, sections were stained with Prussian blue, mixing 1% potassium ferrocyanide with 1% HCl for 8 hours at 37℃. Then sections were washed in 0.01M PBS and treated with DAB solution for 5 minutes. PBS, phosphate buffered saline. Low (a-d) and High-power (e-h) view of histological sections from dentate gyrus showing iron levels were increased in wild type and *Ftmt* knockout mice after injecting $A\beta_{25-35}$ and the highest amount of iron was observed in KO + $A\beta_{25-35}$ group. In addition, the iron level was also increased in the KO + Saline group when compared to the WT + Saline group.

References

1. Meguro, R., et al., *The presence of ferric and ferrous iron in the nonheme iron store of resident macrophages in different tissues and organs: histochemical demonstrations by the perfusion-Perls and -Turnbull methods in the rat.* Arch Histol Cytol, 2005. **68**(3): p. 171-83.
2. Duce, J.A., et al., *Iron-export ferroxidase activity of beta-amyloid precursor protein is inhibited by zinc in Alzheimer's disease.* Cell, 2010. **142**(6): p. 857-67.
3. Falangola, M.F., et al., *Histological co-localization of iron in Abeta plaques of PS/APP transgenic mice.* Neurochem Res, 2005. **30**(2): p. 201-5.
4. Meguro, R., et al., *Nonheme-iron histochemistry for light and electron microscopy: a historical, theoretical and technical review.* Arch Histol Cytol, 2007. **70**(1): p. 1-19.
